# Shorter-lived neural taste representations in obese compared to lean individuals

**DOI:** 10.1038/s41598-018-28847-3

**Published:** 2018-07-23

**Authors:** Samyogita Hardikar, Raphael Wallroth, Arno Villringer, Kathrin Ohla

**Affiliations:** 1 Department of Neurology, Max Planck Institute for Human Cognitive and Brain Sciences, Leipzig, Germany; 2 Psychophysiology of Food Perception, German Institute of Human Nutrition Potsdam-Rehbruecke, Nuthetal, Germany; 3 NutriAct-Competence Cluster Nutrition Research Berlin-Potsdam, Berlin, Germany; 4 Department of Cognitive Neurology, University Hospital Leipzig, Leipzig, Germany; 5 Cognitive Neuroscience, Institute of Neuroscience and Medicine (INM-3), Research Center Jülich, 52428 Jülich, Germany

## Abstract

Previous attempts to uncover a relation between taste processing and weight status have yielded inconclusive results leaving it unclear whether lean and obese individuals process taste differently, and whether group differences reflect differential sensory encoding or evaluative and reward processing. Here, we present the first comparison of dynamic neural processing as assessed by gustatory evoked potentials in obese and lean individuals. Two supra-threshold concentrations of sweet and salty tastants as well as two sizes of blue and green squares were presented to 30 lean (BMI 18.5–25) and 25 obese (BMI > 30) individuals while recording head-surface electroencephalogram (EEG). Multivariate pattern analyses (MVPA) revealed differential taste quality representations from 130 ms until after stimulus offset. Notably, taste representations faded earlier and exhibited a reduced strength in the obese compared to the lean group; temporal generalization analysis indicated otherwise similar taste processing. Differences in later gustatory response patterns even allowed decoding of group membership. Importantly, group differences were absent for visual processing thereby excluding confounding effects from anatomy or signal-to-noise ratio alone. The latency of observed effects is consistent with memory maintenance rather than sensory encoding of taste, thereby suggesting that later evaluative aspects of taste processing are altered in obesity.

## Introduction

Obesity has been associated with altered perception of food cues, with the literature focusing primarily on visual food stimuli (see^1,2^ for review). Several psychophysiological studies using either pictorial or verbal cues have pointed towards an attentional bias and augmented sensitivity to food cues in obese compared to lean individuals, irrespective of the technique used^1^. This is also corroborated with neuroimaging, as obese compared to lean participants are found to have higher blood oxygen level dependent (BOLD) responsivity to food cues, especially for high calorie foods^3,4^.

Comparatively fewer studies have investigated the role of body weight in taste processing. This is surprising given its pivotal role in nutrient sensing - gustation facilitates decisions as to the edibility and spoilage of food - and food-related behaviour. The few findings regarding gustatory perception are also more ambiguous. For instance, obese compared to lean participants have been found to display a lower sensitivity to all four tastes^5^, a higher sensitivity to sweet and salty^6^, or no difference in taste sensitivity^7^. Seemingly mixed is the evidence from weight-loss intervention studies: higher taste sensitivity after surgery-induced weight loss was reported when using the taste-strips method^8^ while taste sensitivity was unaffected in surgery-induced weight loss when measured as sensory thresholds to sapid tastants^9,10^. Some of the discrepancy may be explained by the complex nature of ingestive behaviour, the multitude of variables under investigation, and the heterogeneity of methods used for taste sensitivity assessment. Additionally, achieving chemosensory stimulation in a precise and controlled way is more challenging than visual, auditory, or somatosensory stimulation. It is partly due to this reason that researchers so far have relied greatly on verbal or visual food stimuli, rather than real foods or gustatory stimuli. Especially neuroimaging investigations of taste and obesity are few in number and present an inconclusive picture. Functional magnetic resonance imaging (fMRI) studies have reported a BMI-dependent higher BOLD response in the rolandic operculum^11–13^, and the insula^11,12^ - both areas of sensory taste representation^14,15^. Whereas recently Frank *et al*.^16^ reported a lower taste discrimination in the insula in obese compared to lean.

The evidence is even more complicated where the evaluative or reward related brain areas are concerned. When a palatable food stimulus (milkshake) was used, adolescent females with obesity showed a higher anticipatory reward response to the stimulus cue, but a lower striatal reward response on receipt^11^. This attenuation of consummatory food reward in the obese may be mediated by the Taq|A1 gene^17^. On the contrary, Szalay *et al*.^12^ observed a higher BOLD response not only in the insula, operculum, and the OFC - the site of gustatory hedonic encoding - but also subcortical structures like the amygdala and nucleus accumbens in response to both pleasant and unpleasant tastes.

Together, the fMRI literature suggests differential activation of brain areas implicated in gustatory processing, particularly at anatomically early levels within the primary gustatory cortex, the insula and opercula. While such activation may implicate the initial, sensory activation, it could also reflect later, evaluative processes possibly through feedback from higher cortical areas. Insular activation has been implicated in both, sensory processing, e.g. in taste intensity^18,19^ and quality perception^20^ within only 200 ms of taste stimulation, and later evaluative processing, with top-down modulation from cross-modal cues^21^. Due to its poor spatial resolution, fMRI cannot distinguish between these explanations.

In order to elucidate the spatio-temporal dynamics of neural processes, and disentangle the potential differences between lean and obese groups in various stages of processing, a temporal resolution in the millisecond range, as that provided by electroencephalography (EEG), is required. To date, EEG studies of taste perception have been exceptionally rare due to the difficulties involved achieving taste stimulation in a way that is both temporally and quantitatively precise, and not confounded by oral somatosensation^22^. Here we present the first ever exploratory investigation of the neural response to taste between lean and obese individuals as measured by EEG and analysed using multivariate pattern analysis (MVPA).

## Methods

### Participants

55 healthy adults between 18–35 years (25 men, 30 women) participated in the study, 30 of whom were lean (BMI range: 18.5–25 kg/m^2^) and 25 obese, (BMI ≥ 30 kg/m^2^). Participant characteristics are detailed in Table 1. Participants were screened via telephone interviews to exclude those with self-reported taste/smell disorders, smoking, alcohol/other addiction, stroke, depression, diabetes, hypothyroidism, oral/nasal/sinus infections, pregnancy, or recent (last 6 months) childbirth. To minimise the confounding effects of hormonal changes, only women using oral contraceptives were included. All participants gave written informed consent prior to the experiment and were free to quit the experiment at any point without giving a reason. The experimental procedures were in accordance with the Declaration of Helsinki and approved by the medical ethics committee of the University of Leipzig.

**Table 1:** Participant Characteristics (group means and SD).

	Lean (n = 30)	Obese (n = 25)	p-value
Age (years)	25.47 ± 3.81	26.64 ± 3.52	0.245^a^
Sex (men, women)	15, 15	10, 15	0.639^b^
BMI (kg/m^2^)	22.13 ± 1.83	35.48 ± 4.53	<0.001^a,c^

^a^Two-sample t-test.

^b^Pearson’s chi-squared test.

^c^Levene’s test is significant suggesting unequal variances.

### Stimuli

Tastants included sucrose (Sigma-Aldrich, CAS number: 57-50-1) and sodium chloride (NaCl; Sigma-Aldrich, CAS number: 7647-14-5) that were dissolved in mineral water (Volvic, Danone Waters Deutschland GmbH), which was also used as rinse. We created four different taste stimuli: high (0.29 M; 10 g/100 mL) and low (0.15 M; 5 g/100 mL) sweet, and high (0.43 M; 2.5 g/100 mL) and low (0.21 M; 1.25 g/100 mL) salty. Volvic mineral water has a mineral content of 130 mg/L (Calcium 12 mg/L, Chloride 15 mg/L, Sodium 12 mg/L, Potassium 6 mg/L, Silica 32 mg/L, Hydrogencarbonate 74 mg/L, Magnesium 8 mg/L, Sulphate 9 mg/L), and a pH of 7.

Visual stimuli were green and blue squares of two sizes (large: 11.9°; small: 6.0°). As this is the first study of gustatory event related potentials (ERPs) in lean and obese individuals, the visual stimuli were included as a control for the analyses of “group” (lean, obese) differences in ERPs. Any difference in visual processing between groups would indicate differential signal-to-noise levels in the recordings or anatomical differences that would then have to be considered in the interpretation of any group difference in taste processing.

### Experimental procedure

Taste stimuli were presented as atomized aliquots of 210 µL delivered over 900 ms through a computer-operated gustometer (GU002, Burghart Messtechnik, Wedel, Germany; see^18,20,23^). Stimuli were embedded into a continuous 3.3 Hz sequence of water sprays to minimize oral-somatosensory responses. Visual stimuli were presented on the center of a computer screen for 900 ms on a grey background.

Participants were asked to sit in front of a computer screen (67 cm from the eyes) leaning forward against the headrest of the gustometer, and extend the anterior half of the tongue under its spray nozzle, which was positioned approx. 1.5 cm above the extended tongue.

**Figure 1:**
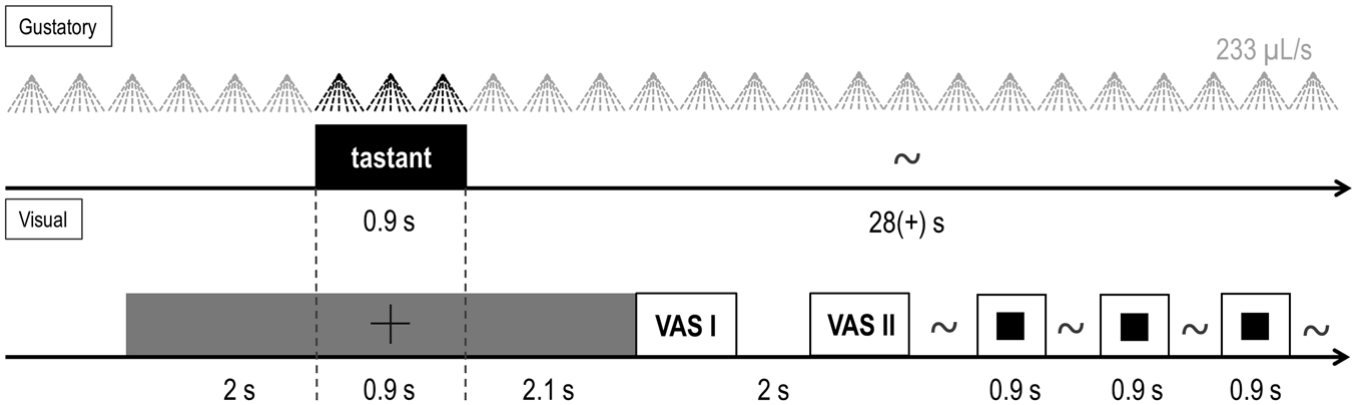
Schematic diagram of an experimental trial. tTop row: spray pulses of the gustometer, tastants are represented in black, water is represented in grey. Bottom row: VAS I and VAS II were visual analog scales for rating stimulus intensity and pleasantness. Filled black squares represent instances of visual stimulus presentation. Variable intervals between events are indicated with ~.

During each trial, a taste and either none, one, two or three visual stimuli were presented sequentially (see Fig. 1 for the schematic of a single trial). On each trial, a central fixation cross appeared on the screen for 2000 ms before a taste stimulus was presented via the spray nozzle for 900 ms; the fixation remained on screen during taste stimulus delivery and 2100 ms thereafter. Next, participants were presented with an on-screen visual analog scale (VAS) to rate the intensity of the taste from 0 (no sensation) to 100 (extremely strong), followed 2 s later by another VAS to rate the pleasantness from 50 (extremely unpleasant) to 0 (neutral) to 50 (extremely pleasant). For this, participants moved a mouse cursor along the scale and logged their rating by clicking the left button. The inter-stimulus interval (ISI) between tastes varied depending on the participants’ response time for the VAS, but a minimum waiting period of 28 s was maintained between trials. During most ISIs, participants were sequentially presented up to three visual stimuli, one at a time, on the computer screen for 900 ms. No task was to be performed with the visual stimuli.

Each of the four taste stimuli was presented 48 times on average. The number of taste trials was increased from 36 to 50 after the first 10 participants to improve the signal to noise ratio of the gustatory response. The order of taste stimuli was counterbalanced, such that the same taste quality was never presented on consecutive trials.

Each of the four visual stimuli was presented 65 times. The number of visual stimulus presentations during each ISI varied between zero and three, and the order of presentation was randomised.

The experiment was divided into 10 blocks of 11–12 min. Blocks were separated by breaks of two to five minutes as needed and requested by participants. On average, the experiment lasted 120–140 minutes, and an additional 45–60 minutes were required for preparation and training. Participants went through a short training (three trials) prior to the experiment to get acquainted with the procedure and to find a comfortable posture and tongue position that could be held for the duration of a block. They were instructed to hold the tongue in the same position for the duration of the block and, if absolutely necessary, to move it only immediately after the VAS presentations, to avoid movement during epochs of interest and to have a substantial “spray-habituation” period before the next trial. The stimulation required no swallowing.

Transitor-transitor logic (TTL) pulses between the gustometer and the stimulation and EEG computers controlled the timing of stimulus onset, and also logged it into the EEG recording files. The delay between these pulses prompting the syringe plungers of the gustometer to push liquids through individual Teflon® tubes and the spray nozzle, and the atomized liquids reaching the tongue is 36 ms (SD = 2 ms) with a rise time to reach 70% of the maximum response of <15 ms (data provided by the manufacturer based on an identical setup). The separate tubes carrying the tastants and water to the spray nozzle were enveloped by a hose of warm water (39 °C), and the pressure of the spray was kept constant throughout.

### EEG acquisition and pre-processing

Participants were seated in a sound-attenuated recording booth during the experiment and the gustometer was positioned outside the booth to minimize acoustic disturbance from the device. Head-surface EEG was continuously recorded with the actiCHamp amplifier system (Brain Products GmbH, Munich, Germany) from 62 silver/silver chloride (Ag/AgCl) active electrodes mounted in an elastic cap according to the extended 10–10 system; another electrode was placed under the left eye to monitor eye movements. FCz served as reference during recording only. The EEG was recorded with BrainVision Recorder Professional V. 1.20.0506 (Brain Vision LLC, Morrisville, NC, USA) at 500 Hz using analogue filters (0.01 Hz high-pass and 200 Hz low-pass). The continuous EEG data were processed offline by using custom-made scripts in MATLAB (MathWorks, Natick, MA, USA) and the EEGLAB toolbox^24^. Malfunctioning channels were interpolated manually before re-referencing data to average of all channels. The continuous data were low-pass filtered with a 44 Hz cut-off and 8 Hz transition width (order 208) and then high-pass filtered with a 0.1 Hz cut-off (order 8250) using a zero-phase Hamming windowed sinc FIR filter with a maximum passband deviation of 0.2% and a stopband attenuation of −53dB (cf.^25^). Data were then segmented into epochs of 2 s with an additional 500 ms pre-stimulus baseline period. Epochs with unique recording artefacts were rejected by visual inspection. Further, commonly observed artefacts (e.g. ocular, muscular, or vascular) were identified using Infomax independent component analysis as implemented in EEGLAB^26^ and removed. Overall <2% of all trials were rejected. Epochs were separately analysed for gustatory and visual trials.

### Statistical Analyses

First, global measures of evoked field strength and distribution were compared in order to get a general overview of evoked neural gustatory and visual responses and to verify that we have above baseline gustatory activation. The instantaneous topographical patterns of evoked responses were then explored further using multivariate pattern analysis on a single-trial level to see whether taste-quality information emanated differently in the lean and obese groups, and whether these two groups could be differentiated based on their neural response patterns to the same taste stimuli.

### Perceptual Ratings

VAS ratings of taste intensity and pleasantness were aggregated across trials for each participant and for each condition and submitted to a repeated measures analysis of variance (ANOVA) with “taste quality” (salty, sweet) and “taste concentration” (high, low) as the within-subject factors, and “group” (lean, obese) as the between-subjects factor. The alpha level was set to 0.05 for all statistical analyses if not specified otherwise. Non-significant effects from the ANOVA were followed up with Bayesian independent samples t-tests in JASP^27^ to estimate whether the data are merely inconclusive or strongly in favour of the null hypothesis.

### Global Field Power (GFP) and Global Map Dissimilarity (GMD)

To visualize the overall EEG activity, GFP, a reference-free index of overall field strength^28,29^, was calculated. GFP is analogous to the standard deviation over the entire electric field (all electrodes) at each sampling point and was calculated in Ragu (Randomization Graphical User interface^30^) using the following formula:

1 $${\bf{G}}{\bf{F}}{\bf{P}}=\,\sqrt{\frac{{\sum }_{{\boldsymbol{j}}=1}^{{\boldsymbol{n}}}({{\boldsymbol{v}}}_{{\boldsymbol{j}}}-\bar{{\boldsymbol{v}}})}{{\boldsymbol{n}}}},$$

where *v*_*j*_ is the voltage measured at sensor *j*, *n* is the number of sensors, and $\bar{v}\,\,$is the mean measurement across all sensors^30^. GFP was calculated for each participant and for each condition separately. To explore differences between experimental conditions, post-stimulus GFPs were submitted to a 3-way ANOVA with “taste quality” (salty, sweet) and “taste concentration” (high, low) as the within-subject factors, and “group” (lean, obese) as the between-subjects factor.

GMD^31^ between conditions and groups was calculated as an index of differences in the scalp field (topography) generated by all electrodes, using the following formula:

2 $${\bf{G}}{\bf{M}}{\bf{D}}=\,\sum _{{\boldsymbol{i}}=1\,}^{{\boldsymbol{c}}}\sqrt{\frac{{\sum }_{{\boldsymbol{j}}=1}^{{\boldsymbol{n}}}{({\bar{{\boldsymbol{v}}}}_{{\boldsymbol{ij}}}-{\overline{\overline{{\boldsymbol{v}}}}}_{{\boldsymbol{j}}})}^{2}}{{\boldsymbol{n}}}},$$

where *c* is the number of conditions and group, *n* is the number of sensors, ${\bar{v}}_{ij}$ is the voltage of the grand mean across subjects of condition and/or group *i* at sensor j, and ${\overline{\overline{v}}}_{j}$ is the grand mean across subjects and conditions of the voltage at sensor *j*^30^. Importantly, in order to calculate GMD independent of field strength, all data were normalised prior to the GMD calculation as recommended by Koenig and colleagues^30^, using the L2-norm (least squares) function provided in Ragu^30^ which sets all data to equal variance across all electrodes before analysis^30^. Post stimulus GMD was analysed in a topographical-ANOVA (t-ANOVA) with the same factors as above with 5000 permutations using Ragu^30^.

As no significant effects of “taste concentration” were observed for either the GFP or GMD, the data was collapsed across the two taste concentrations, and the 3-way ANOVA was reduced to “taste quality” (sweet, salty) × “group” (lean, obese). Similarly, the GFP and GMD for the visual evoked responses were calculated with a “colour” (green, blue) × “group” (lean, obese) ANOVA. Duration thresholds for significant effects were calculated (see^30^) and applied to results from the ANOVA to correct for multiple comparisons across time.

### Multivariate pattern analysis (MVPA)

To test for the emergence of taste quality and intensity information in the EEG signal at the single trial level, linear support vector machine (SVM) classifiers^32^ were trained and tested at each time point in a sliding time window approach. This procedure is generally referred to as multivariate pattern analysis (MVPA, cf.^33^), where the machine learning algorithm attempts to leverage predictive information with respect to the stimulus class from an instantaneous topographical pattern of the amplitudes of all electrodes. For the discrimination of stimulus category MVPA was conducted separately for lean and obese participants. Trials were then pooled across participants in order to maximize generalizability. Consequently, the cross validation (CV) schemes, which separate the data into folds of training and testing sets, were stratified for both, the stimulus class and the participants, such that the trial composition of each fold was a balanced reflection of the sample distribution. This splitting of data into training set (data the classifier learns with) and testing set (data the classifier is tested on) is commonly done in order to obtain unbiased performance estimates of the classifiers. To attenuate individual differences, we scaled all trials with the mean and standard deviation of their respective baseline periods. The baseline period was set to 200 ms up to stimulus onset for taste and 100 ms up to stimulus onset for the visual condition. To improve the signal-to-noise ratio, EEG data were re-sampled to 100 Hz and the taste data additionally low-pass filtered (−6 dB cut-off 5.5 Hz, transition width 1 Hz, order 330) using a zero-phase Hamming windowed sinc FIR filter with a maximum passband deviation of 0.2% and a stopband attenuation of −53 dB (cf.^25^) because taste information has been previously shown to be limited to this particular frequency range^34^. An L2-regularization with a C parameter value of 10^−4^ was set for the SVM classifiers to enforce small weights and greater stability. Classifier performance was estimated at each time point using the area under the receiver operating characteristic curve (AUC), a balanced accuracy metric where 50% corresponds to chance performance even with uneven class distributions. All MVPA analyses were implemented with custom scripts in R^35^, using the ‘LiblineaR’ library for the SVM algorithm^36^.

#### Classification of taste quality

Decoding of taste quality information was implemented with a 20-fold stratified CV, where the classifiers were iteratively trained on a randomly selected subset of 95% of the trials and tested on the independent subset of the remaining 5% of trials. Data of lean and obese participants were pooled and analysed separately to compare the classification performance between the two populations. SVM classifiers were trained to make the binary discrimination between sweet and salty taste (irrespective of the concentration) and, for the visual control condition, between green and blue squares (irrespective of stimulus size). Differences between the classification performances of lean and obese were assessed at each time point for each modality with two-sided Wilcoxon rank sum tests; significant *p*-values (<0.05) were adjusted to a minimum duration of 100 ms. Significant decoding of category (within each group) was evaluated with one-sided binomial tests which assert above-chance performance while considering sample size.

#### Classification of group membership

In order to test whether lean and obese individuals can be discriminated given taste-related neural response patterns within a single category, classifiers were trained separately for each stimulus category irrespective of concentration or size (i.e. sweet, salty, green, blue). Specifically, a 10-fold stratified CV was adapted to learn generalizable patterns associated with one or the other group based on the instantaneous topographical distribution for a given trial (i.e. tasting sweet; viewing green). To disentangle group differences in gustatory processing from those unrelated to gustatory processing (e.g. due to anatomy or signal-to-noise ratio) we repeated the analyses over 1000 permutations of group membership and compared the best performing permutation, an optimistic estimate of irrelevant differences, with the observed classification performance. The permutations were constrained to be unique and within a margin of 12.5% of a perfect shuffle (i.e. when exactly half of the labels, lean and obese, were exchanged) and applied to each stimulus category. Taste-evoked neural response patterns differ between lean and obese where permutation thresholds are exceeded for the duration of at least 100 ms. The difference between the actual and the maximum permutation performance provides a direct estimate of the effect size.

#### Generalization across time and groups

To characterize the temporal dynamics of taste information processing and its relation between the lean and obese groups, we applied a generalization method^37^, which is an extension of the common MVPA, separately for both groups (with a 20-fold stratified CV and pooling across participants). Previously, a classifier was trained at one time point and tested at that very same time point. In contrast, here the learned pattern is applied at all time points irrespective of where it was observed originally. This approach is useful to see how far the neural response pattern at a certain time point generalizes backward and forward in time, enabling the examination of correlated activation clusters. If a pattern generalizes over longer time periods one can conclude sustained activation in response to the stimulus, whereas shorter but multiple temporal clusters suggest different, independent processing steps in response to a stimulus.

The decoding result provides a matrix whose cells hold the AUC of a combination of training and testing (generalization) time, one per group. The diagonal of the generalization matrix represents the MVPA with training and testing conducted at the same time point. Performance increases along the horizontal and vertical dimensions reflect sustained neural responses because neural patterns at one time point resemble those at earlier and later time points. In contrast, performance increases limited to the main diagonal reflect distinct neural patterns at a given time. Differing dynamics in gustatory processing between groups would result in significant differences for the contrast of the two generalization matrices. Two-sided Wilcoxon rank sum tests with false discovery rate (FDR^38^) adjustment were used to evaluate statistical significance. To verify above- or below-chance generalizability across time (within one group) and differences between main- and off-diagonal performance, two-sided binomial tests were conducted.

### Data availability

The datasets generated and analysed during the current study are available from the corresponding author on reasonable request.

## Results

### Perceptual Ratings

As expected, participants rated lower concentrations as less intense (F_1,53_ = 175.449, p < 0.001, η² = 0.768) and preferred the lower concentration of a taste over the higher one (F_1,53_ = 51.454, p < 0.001, η² = 0.492). Moreover, sweet was preferred over salty (F_1,53_ = 108.248, p < 0.001, η² = 0.671) and salty was rated more intense than sweet (F_1,53_ = 61.259, p < 0.001, η² = 0.534). Lean and obese participants rated all tastes similarly (all F < 0.9, all p > 0.05). Perceptual ratings are summarized in Table 2.

**Table 2:** Perceptual ratings.

Quality	Conc.	Group	n	Intensity		Pleasantness	
				Mean	SD	Mean	SD
Sweet	High	LN	30	53.54	18.10	7.132	7.727
		OB	25	51.07	19.37	7.124	15.174
	Low	LN	30	42.55	20.35	9.824	6.276
		OB	25	40.94	19.83	9.576	14.551
Salty	High	LN	30	67.43	14.86	−15.908	10.027
		OB	25	68.17	11.54	−16.353	11.357
	Low	LN	30	59.23	15.73	−11.116	9.435
		OB	25	59.48	12.16	−10.645	10.181

LN = Lean, OB = Obese.

As no significant effect of group was apparent, Bayesian independent samples t-tests were conducted for all ratings in order to ascertain whether the data are merely inconclusive or strongly in favour of the null hypothesis regarding the effect of “group”. All Bayes factors (BF_01_) had values between 3.318 and 3.365, suggesting that the evidence in favour of the null hypothesis (i.e. no difference in perceptual ratings between the lean and obese group) is not particularly strong.

### GFP and GMD

#### Gustatory

**Figure 2:**
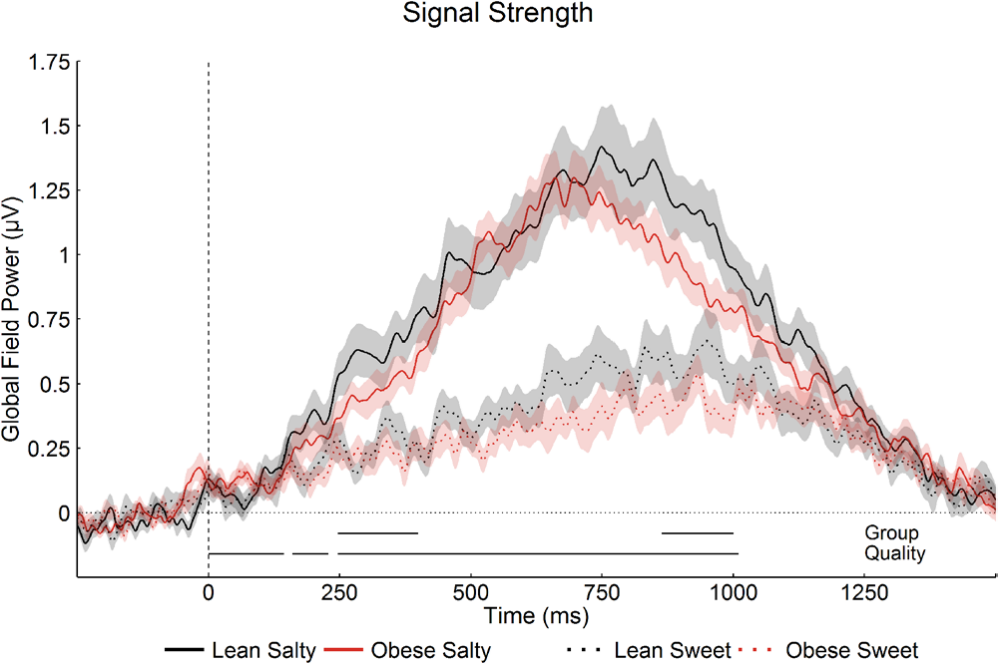
Global Field Power (Gustatory). Mean GFP (±1 SEM indicated as shaded area surrounding the mean) for sweet and salty tastes, in lean and obese groups (baseline removed for visualisation only). The dashed vertical line indicates stimulus onset. Stimuli were presented for 900 ms. Post-stimulus periods of significant effects (p < 0.05, >44 ms for “Quality”, p < 0.05, >64 ms for “Group”) are marked by horizontal black bars above the x-axis.

GFP was significantly higher for salty than for sweet at each point from 0 ms to 1010 ms post stimulus onset (p < 0.05, >44 ms), and significantly higher in the lean than in the obese group from 246 ms to 400 ms and from 864 ms to 1000 ms (p < 0.05, >64 ms). GFP for the two taste qualities and groups is shown in Fig. 2.

Electric field distributions, compared using GMD, did not differ significantly between groups (p < 0.05, >54 ms) and no significant group*quality interaction was observed.

#### Visual

**Figure 3:**
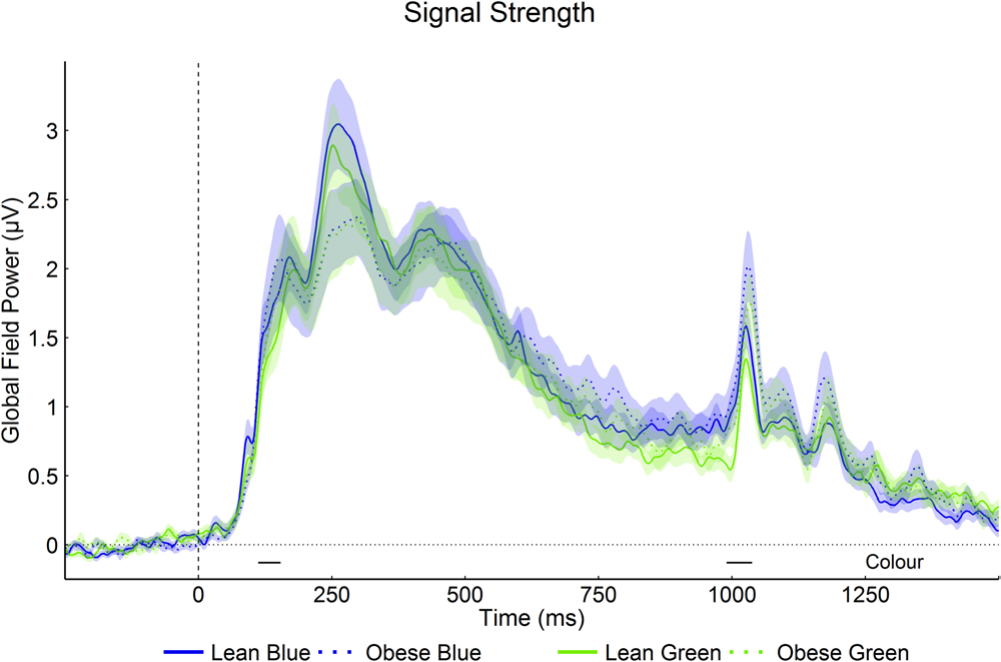
Global Field Power (Visual). Mean GFP (±1 SEM) for green and blue squares, in lean and obese groups (baseline removed for visualisation only). The dashed vertical line indicates stimulus onset. Stimuli were presented for 900 ms. Post-stimulus periods of significant effects (p < 0.05, >34 ms) are marked by horizontal black bars above the x-axis.

GFP was significantly higher for blue than for green squares 112 ms to 154 ms after stimulus onset (from 112 ms to 154 ms; p < 0.05, >34 ms) and stimulus offset (from 990 ms to 1038 ms; p < 0.05, >34 ms). GFPs did not differ significantly between groups and no significant group * colour interaction was observed. GFP for the two colours and groups is shown in Fig. 3.

Electric field distributions differed significantly between green and blue from 100 ms to 154 ms, 446 ms to 496 ms, 506 ms to 558 ms, and 602 ms to 690 ms after stimulus onset (p < 0.05, >28 ms). Significant map dissimilarities differences were observed between the lean and obese groups shortly after stimulus offset (from 1034 ms to 1096 ms; p < 0.05, >54 ms) only. No significant group *colour interaction was found.

### MVPA

#### Classification of taste quality

**Figure 4:**
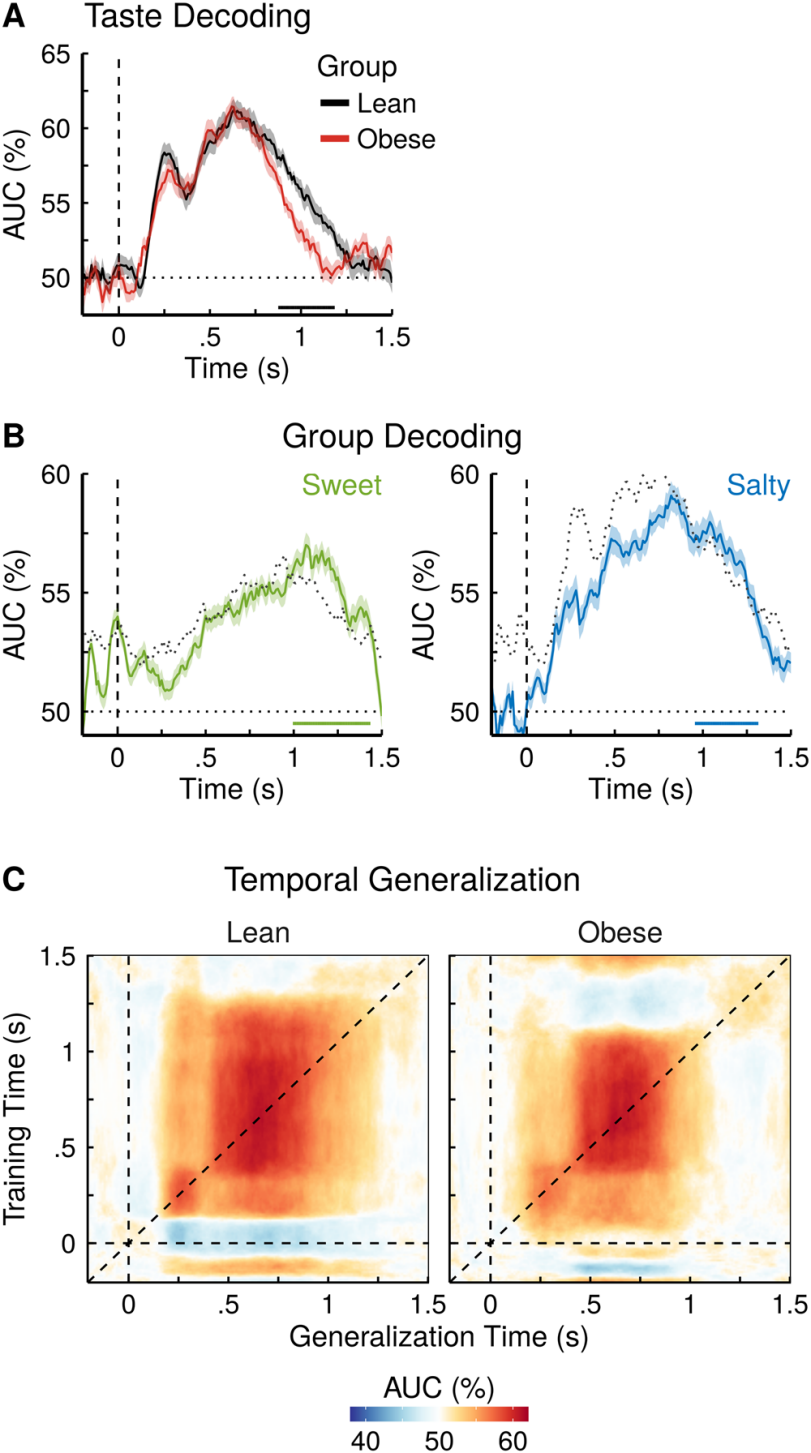
Decoding of taste information, group membership and temporal generalization reveals differences in taste processing between lean and obese individuals. (**A**) Binary decoding of taste quality information (salty versus sweet), separately for lean and obese individuals. Coloured solid lines show the mean performance, surrounding shaded regions show 1 SEM (dashed vertical line: stimulus onset; dotted horizontal line: theoretical chance level of an AUC of 50%). Time points with significant group differences (*p* < 0.05) are indicated by a horizontal black bar above the x-axis. (**B**) Binary decoding of group membership within a taste quality (e.g. given a sweet taste, “is this a lean or an obese person?”). The jittered black line shows the maximum performance over 1000 permutations of group membership per time point. The coloured horizontal bars above the x-axis indicate time points where the actual performance curve exceeded the permutation threshold (minimum of 100 ms). (**C**) Generalization across time. The diagonals of the matrices (same training and testing time) correspond to the performance curves in A. Square generalization patterns (i.e. symmetric increases along the vertical and horizontal dimensions) suggest sustained activity that generalizes across time.

**Figure 5:**
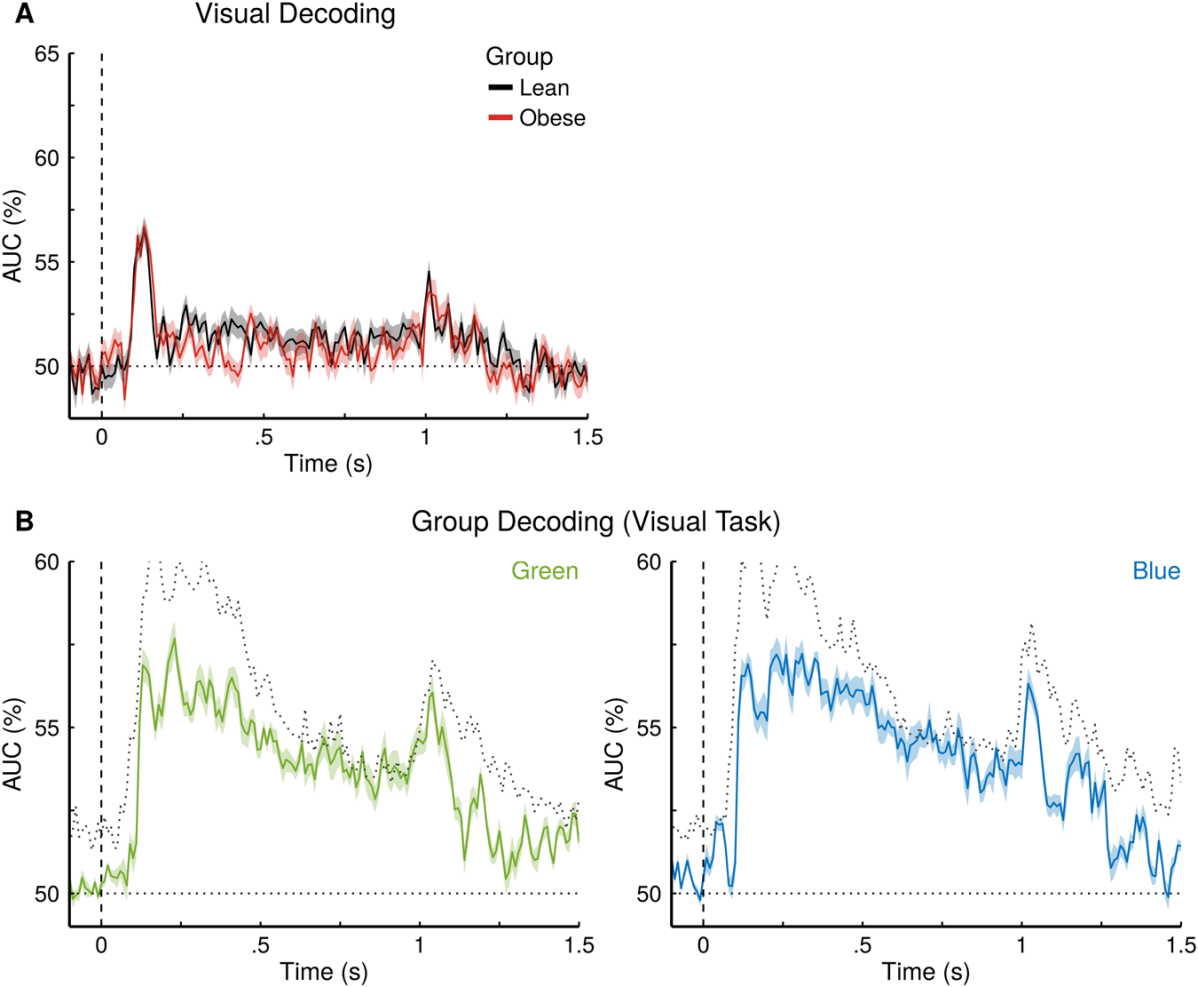
Decoding of visual information and group membership reveal no differences in visual processing between lean and obese individuals. (**A**) Binary decoding of colour information (green versus blue), separately for lean and obese individuals. Coloured solid lines show the mean performance, surrounding shaded regions show 1 SEM (dashed vertical line: stimulus onset; dotted horizontal line: theoretical chance level of an AUC of 50%). (**B**) Binary decoding of group membership within a colour (e.g. given a green square, “is this a lean or an obese person?”). The jittered dark grey line shows the maximum performance over 1000 permutations of group membership per time point.

Taste quality information emerged rapidly in the neural response patterns after stimulus onset (Fig. 4A), reaching statistical significance at 150 ms for lean (AUC = 51.2% +/−0.6, *p* = 0.041) and at 130 ms for obese subjects (AUC = 52.0% +/−0.6, *p* = 0.003). Decoding accuracy remained continuously significant until 1260 ms for lean (AUC = 51.5% +/−0.8, *p* = 0.013) and 1100 ms for obese subjects (AUC = 51.5% +/−1.0, *p* = 0.026), clearly outlasting taste stimulation. Significant group differences (in favour of lean) between the classification performances emerged during later stages of taste processing, starting at 880 ms (difference = 1.8% +/−0.9, *p* = 0.049), until 1180 ms (Difference = 2.7% +/−0.6, *p* = 0.006). The onset of this difference coincides closely with the transition from taste stimulation to rinsing (stimulation offset 700 ms, rinsing onset 900 ms). No group differences were observed for the visual control task in which the classifiers discriminated between blue and green squares (Fig. 5A).

#### Classification of group membership

The classification of group membership (Fig. 4B) was found to exceed chance performance for an extended period of time for both tastes (1000 ms to 1430 ms for sweet and from 960 ms to 1310 ms for salty taste). The effect lasted longer and was more pronounced for sweet (peak: 1200 ms, 2.2% above threshold) compared to salty (peak: 1170 ms, 1.4% above threshold). No such persistent group differences were found for the MVPA applied to the visual control task (Fig. 5B).

#### Temporal Generalization

The results showed a square pattern of generalization across time for both groups (Fig. 4C), suggesting on-going, interdependent mental activity with respect to taste processing. A smaller first time window of generalization was identified within the first 400 ms after stimulus onset (peaks: lean 270 ms, AUC = 58.4% +/−0.7; obese 290 ms, AUC = 57.6% +/−0.9), whose generalization performance did not differ significantly to the peak testing time from 210 to 340 ms for lean and 220 to 400 ms for obese subjects (*p* > 0.05, uninterrupted). A second, larger time window of generalization was found with its peak at 630 ms for lean (AUC = 61.4% +/−0.6) and 610 ms for obese subjects (AUC = 61.0% +/−0.7), with no significant difference between diagonal and off-diagonal performance from 510 to 820 ms (lean) and 470 to 800 ms (obese; *p* > 0.05, uninterrupted). The latter window’s peak time patterns then generalized significantly until 1230 ms and 1090 ms for lean and obese, respectively (*p* < 0.05, uninterrupted). This finding suggests that taste processing is rapidly initiated by a shorter, likely purely perceptual state before transitioning to a longer evaluative phase. Nevertheless, the first process is of relevance to the second one as its neural patterns generalize significantly until 1150 ms and 990 ms for lean and obese, respectively (*p* < 0.05, uninterrupted). A comparison of the temporal generalizability in the two groups revealed that in the lean group, the neural patterns from the time window between 1100 and 1260 ms (offset of decodability for obese and lean, respectively) generalized significantly better from ~300 up to 1080 ms (p_FDR-corrected_ < 0.05). In other words, the second step in the processing chain which starts around 300 ms is prolonged for lean as compared to obese. Consequently, this means the temporal generalization reveals the same number of processing steps for both groups, but with differing durations.

## Discussion

In the absence of a clear account of differences in taste perception and their neural underpinnings in obesity, it is important to look at the whole perceptual process, and to disentangle the sensory and evaluative aspects wherever possible. Thus, in the present study, we analysed gustatory evoked responses in lean and obese individuals with the help of EEG, which offers temporal resolution on a millisecond time scale. We employed electro-physiological measures such as evoked field strength and topographical distribution, as well as multivariate pattern analysis which can provide avenues for further inspection even when the signal to noise ratio is not optimal.

In line with previous findings, taste quality information emerged shortly after the stimulus onset^20^ and was present even after the end of stimulation in both the lean and obese groups. Remarkably, taste quality information deteriorated earlier in the obese than the lean group, suggesting that lean and obese individuals display differences during the later stages of taste processing. In line with this finding, for both taste qualities, group-membership decoding was possible only during this later stage further corroborating that the time period around taste offset differentiates between groups. Analysis of temporal generalization indeed points to similar processes between groups that vanish earlier in the obese, rather than an additional processing step in the lean. This interpretation is also supported by the lower GFP in obese compared to lean group close to the stimulus offset, and the lack of corresponding global map dissimilarities as seen in the GMD analysis. Overall, these findings suggest consistently that lean and obese individuals process taste quality similarly, yet obese individuals exhibit shorter lasting activity within the gustatory network.

Also in line with previous findings, the GFP for salty was significantly higher than for sweet, signifying lower SNR for sweet taste^20,23^. Although this difference was already present earlier than would be expected from the latency of the gustatory evoked response, this could be due to the fact that the same taste quality was never presented twice in a row, which may have led to some effect of expectancy.

The latency of group differences in representation seen here is close to stimulus offset, and consistent with working memory maintenance rather than any previously reported sensory or evaluative components of the gustatory evoked potential^15,19–21,23,39^, although this interpretation is purely speculative at this stage. Evidence from the rodent brain shows that gustatory processing takes place via networks of feedback and feedforward pathways that involve more than just the primary sensory areas^40^. Given this and the limited knowledge of the taste processing cascade in humans, we use the term “taste representations” not only with reference to the earliest sensory processing, but in a broader sense. Unlike a previous report^11^ of a lower consummatory food reward to appetitive stimuli in obesity, the group differences reported here were observed for both, pleasant sweet and the somewhat unpleasant salty tastes. It is therefore unlikely that the differences between groups reflect differences in positive reward value per se. Nevertheless, it would be interesting to explore whether the shorter taste representations observed in the obese might be linked to a significantly weaker taste experience over time, perhaps contributing to the reward deficiency seen in obese individuals upon consumption^11^.

Importantly, our experiment included visual control stimuli to exclude that the group differences in gustatory processing resulted from anatomical differences or differences of signal to noise ratio between the two groups. None of the effects seen in the gustatory modality were seen for the visual stimuli. Along with the method of comparing the actual decoding performance against the best performance from 1000 permutations, this presents a strong case that observed differences are in fact related to gustatory processing and cannot be explained by extraneous factors like signal-to-noise ratio alone.

The observed group differences in the electrophysiological signal occurred in the absence of statistically significant self-reported perceptual differences. In an earlier study, we found a heightened sensitivity to sweet and salty taste in obese compared to lean individuals^6^. The present data did not replicate this finding. This apparent discrepancy may be the result of the different method of stimulus administration used in the two experiments (e.g. in the previous study, stimuli were manually sprayed on the tongue with the help of a spray bottle, whereas in the current study, a gustometer was used and the taste stimuli were embedded in a continuous series of water pulses), underscoring the sensitivity of individual differences in taste perception to experimental parameters. Moreover, upon calculation of Bayes factors, the current data do not support the null hypothesis very strongly, thus, underlying perceptual differences between the two groups cannot be ruled out entirely.

Together, we present evidence for differences in gustatory neural processing between lean and obese individuals independent of subjective perceptual differences. Given that this is the first study of its kind, and the ambiguities in the existing literature, more work is needed to investigate the generalizability of the current findings across populations and methodologies. While the underlying biological mechanism behind these differences, as well as their implications for ingestive behaviour remain to be uncovered, the novel combination of electrophysiological data with MVPA offers an avenue into obesity research where the dynamics of gustatory perception and reward may be studied in greater detail.
